# Atypical processing of tones and phonemes in Rett Syndrome as biomarkers of disease progression

**DOI:** 10.1038/s41398-020-00877-4

**Published:** 2020-06-10

**Authors:** Olga V. Sysoeva, Sophie Molholm, Aleksandra Djukic, Hans-Peter Frey, John J. Foxe

**Affiliations:** 1 The Cognitive Neurophysiology Laboratory, Ernest J. Del Monte Institute for Neuroscience, Department of Neuroscience, University of Rochester School of Medicine and Dentistry, Rochester, NY USA; 2 The Cognitive Neurophysiology Laboratory, Departments of Pediatrics and Neuroscience, Albert Einstein College of Medicine & Montefiore Medical Center, Bronx, NY USA; 3 The Laboratory of Human Higher Nervous Activity, Institute of Higher Nervous Activity and Neurophysiology, Russian Academy of Sciences, Moscow, Russia; 4 The Rett Syndrome Center, Department of Neurology, Montefiore Medical Center & Albert Einstein College of Medicine, Bronx, NY USA

**Keywords:** Neuroscience, Prognostic markers

## Abstract

Due to severe motor impairments and the lack of expressive language abilities seen in most patients with Rett Syndrome (RTT), it has proven extremely difficult to obtain accurate measures of auditory processing capabilities in this population. Here, we examined early auditory cortical processing of pure tones and more complex phonemes in females with Rett Syndrome (RTT), by recording high-density auditory evoked potentials (AEP), which allow for objective evaluation of the timing and severity of processing deficits along the auditory processing hierarchy. We compared AEPs of 12 females with RTT to those of 21 typically developing (TD) peers aged 4–21 years, interrogating the first four major components of the AEP (P1: 60–90 ms; N1: 100–130 ms; P2: 135–165 ms; and N2: 245–275 ms). Atypicalities were evident in RTT at the initial stage of processing. Whereas the P1 showed increased amplitude to phonemic inputs relative to tones in TD participants, this modulation by stimulus complexity was absent in RTT. Interestingly, the subsequent N1 did not differ between groups, whereas the following P2 was hugely diminished in RTT, regardless of stimulus complexity. The N2 was similarly smaller in RTT and did not differ as a function of stimulus type. The P2 effect was remarkably robust in differentiating between groups with near perfect separation between the two groups despite the wide age range of our samples. Given this robustness, along with the observation that P2 amplitude was significantly associated with RTT symptom severity, the P2 has the potential to serve as a monitoring, treatment response, or even surrogate endpoint biomarker. Compellingly, the reduction of P2 in patients with RTT mimics findings in animal models of RTT, providing a translational bridge between pre-clinical and human research.

## Introduction

Rett Syndrome (RTT) is a neurodevelopmental disorder caused by a spontaneous mutation in the *MECP2* gene located on the X-chromosome^1^. Its primary clinical features include severe motor deficits and cognitive impairments^2–4^. Verbal ability is typically also very restricted or absent, and combined with the motor impairments, this creates major challenges for clinicians and caregivers in evaluating the degree to which individuals with RTT can understand and differentiate speech or indeed, more basic auditory signals. Direct recordings of the auditory evoked potential (AEP) using high-density electroencephalography (EEG) provide the opportunity to directly study the neurophysiological bases of auditory signal processing in RTT, allowing for objective assessment of the severity of potential auditory processing deficits, with implications for processing of the more complex speech signal. Clinically, these AEP measures may also prove useful as biomarkers of disease severity and progression, as well as targets against which to measure the efficacy of therapeutic interventions during clinical trials.

Early electrophysiological studies in RTT mostly focused on the auditory brainstem response (ABR), with findings indicating that initial subcortical stages of auditory signal processing appeared mostly unaffected^5^. In contrast, AEP studies assessing later cortical stages of auditory processing have tended to show quite significant impairments^5–11^. For example, Stach et al.^5^ reported that more than 50% of individuals with RTT showed atypical AEPs, although they did not precisely specify what these abnormalities were. Others have pointed to delayed auditory processing. For example, Bader et al.^6^ reported significant prolongation of the peak latencies of a series of AEP components (i.e. Pa, N1, and P2), and Stauder et al.^7^ uncovered atypical developmental changes in the latency of the N2. Recent work from our group accords well this notion of slowed auditory processing, as we found substantial delay in the mismatch negativity (MMN) response, an AEP component that is automatically generated when occasional deviant stimuli interrupt a stream of standard stimuli^8^. In that study, we specifically assessed MMN responses to large changes in pitch, and the implications of our results were that cortical representation of even such a basic feature as frequency was atypical and delayed in RTT. Similarly, using the MMN to assess auditory sensory memory for duration, we found that individuals with RTT showed substantial impairment, such that the duration-evoked MMN was only present at rapid stimulation rates (~2 Hz) but was not detectable when slower stimulation rates (circa 0.5 and 1 Hz) were utilized^10^.

While there is some AEP research using frequency-specific tone-pip stimuli in RTT syndrome, AEPs in response to more complex sounds such as speech have not yet been closely examined. Given that a key question in the RTT population is the extent to which non- or minimally-verbal individuals can process and understand the spoken word, there is an imperative to map cortical auditory processing abilities in these individuals. Recent reports by Key et al. suggest that there may be quite profound processing atypicalities for speech tokens. They reported abnormalities in the AEP to speech stimuli in the latency range from 200 to 500 ms post-stimulus when the responses to real words were compared to those to non-words^11^ and from 250 to 450 ms and 450 to 750 ms when AEPs to a participant’s own name were compared to those evoked by other names^9^. However, the work was not designed to assess the earlier stages of cortical processing, during the initial component structure of the AEP, as relatively few trial repetitions were recorded (*n* = 32), which precludes reliable differentiation of earlier AEP components. These initial components are sensitive to stimulus complexity and are implicated in speech processing in children^12–16^. As such, there remains a need to systematically study the initial sequence of AEP components (i.e. P1, N1, P2, and N2) in response to speech tokens, so that the integrity of the successive processing stages along the auditory hierarchy can be examined.

Here, we use high-density electrical mapping of the AEP to probe these processing stages in response to both simple tones and complex phonemic sounds in females with RTT. For this purpose we re-analyzed tone-evoked data from our previous odd-ball study^8^ with a focus on responses to standard stimuli repeated more than 800 times to ensure high signal-to-noise ratios, and thus allow for detailed interrogation of the auditory response.

## Methods

### Participants

Thirteen female patients with Rett syndrome participated in this study (mean age 12.9; range 3.9–20.6). They were recruited during clinical visits to the Rett Center at the Children’s Hospital of Montefiore Medical Center in the Bronx, New York. Diagnosis was based on current diagnostic criteria^4^ and was confirmed clinically by a medical doctor specializing in this population (A.D.) as well as via genetic testing. Symptom severity was assessed for each patient using the Rett Syndrome Severity Scale (RSSS), as modified by Kaufmann et al.^17^. This clinician-rated scale represents an aggregate measure of the severity of clinical symptoms, including motor function, seizures, respiratory irregularities, ambulation, scoliosis, and speech. Each item is scored from 0 (absent/normal) to 3 (severe). Demographic characteristics of the Rett group are summarized in Table 1. RTT participants were compared to a control group of 21 age-matched females (mean age 12.44; range 4.3–21.1) recruited through our database and by posting flyers in the local (East Bronx) community. These typically developing (TD) individuals had no familial history of Rett syndrome and no current or lifetime history of psychiatric, neurodevelopmental, or neurological disorders.TD participants passed a hearing screen and were excluded if they had a biological first-degree relative with a developmental disorder. The study groups were of similar age (*t* (31) = 0.32, *p* = 0.75). This study was approved by the institutional review board of The Albert Einstein College of Medicine (Protocol Reference Number #2011-447). Written informed consent was obtained from parents or legal guardians, and where possible, assent from the patient was also ascertained. All aspects of the research conformed to the tenets of the Declaration of Helsinki.

**Table 1:** RTT cohort characteristics.

RTT	Age	RSSS	Seizures	Ambulation
1	3.9	7	No	No
2	5.1	13	No	Yes
3	6.9*	14	No	No
4	8.6	14	Yes	Yes
5	10.1	12	No	Yes
6	13.0	15	Yes	No
7	13.5	14	Yes	No
8	13.8	16	Yes	No
9	13.9	10	Yes	Yes
10	16.3	19	Yes	No
11	16.9	12	Yes	Yes
12	20.1	6	No	Yes
13	20.6	13	Yes	No

Each participant’s age (years), scores on the Rett Syndrome Severity Scale (RSSS), comorbid seizure diagnosis and ability to walk (ambulation) are listed. Note that RTT participant #3 was excluded from the main analysis due to high amplitude noise in the EEG that precluded derivation of an adequate AEP (indicated by the asterisk).

### Experimental design

Participants sat in a darkened sound-attenuated electrically shielded booth (Industrial Acoustics Company, Bronx, NY), either in a chair/wheelchair or on a parent’s lap, while watching a movie of their choice on a laptop (Dell Latitude E640) with the volume turned off. Auditory stimuli were presented using a pair of speakers (Bose Companion 2 Series II, Multimedia Speaker System) placed behind the laptop. The participants had to stay awake during the experiment and their functional state was monitored by video camera and EEG-activity. If the experimenter noticed signs of drowsiness, she tried to keep the participants alert by entering the room and introducing snack breaks or changing the movies. An oddball paradigm was employed whereby standard and deviant auditory stimuli were presented randomly with a probability of 0.85–0.15, respectively. There were two experimental conditions with simple tones and phonemes, respectively. In the “Tone” condition, stimuli were sinusoidal tones of two different frequencies, 1000 Hz for the standards and 500 Hz for the deviants. Both tones had a duration of 100 ms, a rise and fall time of 10 ms, and an intensity of 75 dB SPL. In the “Phoneme” condition, the phoneme /ba/, served as the standard and the phoneme /da/ served as the deviant. Both were recorded from a female speaker. Phoneme duration was edited to 250 ms, and they were presented at an intensity of 65 dB SPL. A spectrogram of the stimuli can be seen in Supplementary Fig. 1. Here we report the responses to the more frequently presented standard stimuli, comparing them across stimulus conditions (see Foxe et al.^8^ for a report comparing the standards and deviants in an analysis of the MMN). The Rett group completed an average of 9.64 and 9.53 blocks (range 7–11), with each block containing 140 stimuli, which were presented with a stimulus-onset-asynchrony (SOA) of 900 ms, while the control group completed an average of 10.04 and 9.95 blocks (range 10–11), for Tone and Phoneme conditions respectively. Simple tone blocks were mostly presented first, except in the case of one RTT participant and 8 TD cases. Total recording time was about 40 min. Participants were provided breaks. Block order did not influence the AEPs of interest in the TD sample as examined by ANOVA: Type by Condition Order interaction (F(1,19) < 2.47; *p* > 0.13 for all components).

### EEG recordings

Continuous EEG data were recorded using a BiosemiActiveTwo 64 electrode array, analog-to-digital converter, and fiber-optic pass-through to a dedicated acquisition computer (digitized at 512 Hz; DC-to-150 Hz pass-band). Biosemi replaces the ground electrodes that are used in conventional systems with two separate electrodes: common mode sense and driven right leg passive electrode. These two electrodes create a feedback loop, thus rendering them as references.

### Data processing

All EEG processing and analyses were performed in MATLAB (the MathWorks, Natick, MA, USA) using custom scripts and the FieldTripToolbox^18^. Following recording, the continuous EEG was segmented into epochs of 800 ms in length, from −200 to +600 ms post-stimulus latencies. All epochs were arranged by stimulus type and concatenated. Then the data were bandpass filtered 1–20 Hz using zero-phase forward and reverse Butterworth infinite impulse response filter and examined for artifacts. The use of this restricted pass-band was based on two main considerations. First, the spectral content of the initial cortically generated AEP components of primary interest falls within this band^19^. Second, recordings from individuals with RTT are generally quite noisy in the higher-frequency bands due to muscle noise, so limiting the band above 20 Hz allows for acceptance of more trials during signal averaging and consequently better signal-to-noise ratios in the derived responses. It will be of considerable future interest, albeit experimentally challenging, to assess higher-frequency auditory evoked activity, such as the auditory steady state response^20–22^.

Artifact identification was done via *z*-scores and variability measures: all trials that deviated more than two standard deviations from average were removed. Noisy and bad channels were also identified based on the same algorithm and then restored by interpolation from neighboring channels. The AEP of one RTT participant contained an unacceptable level of high amplitude noise that was more than ten times that which was observed in the typical AEP amplitude, and this participant was therefore excluded from further analysis. After artifact rejection, the mean numbers of trials for the RTT and TD groups respectively in the Tone condition were 756 and 804, and in the Phoneme condition 733 and 763 (ranging from 558 to 906). AEPs were calculated separately for each condition by averaging all relevant trails. Epochs were baselined to the 200 ms pre-stimulus interval and re-referenced to the average of TP7 and TP8 sites (which fall over the left and right mastoid regions where the auditory ERPs tends to invert in polarity vis-à-vis the fronto-centrally focused response). The main contributions to the major AEP components (P1, N1, P2, and N2) come from generators in and around primary auditory cortex along the supra-temporal plane and as such, they give rise to predominantly fronto-central scalp distributions. Thus, for peak amplitude analyses here, we focused on the averaged AEPs from the FCz, FC3, and FC4 electrodes.

### Statistical analysis

Amplitudes of the AEP components were averaged across time points within the following characteristic latency windows (see review^23^) and in line with the TD grand-averaged AEPs (P1: 60–90 ms, N1: 100–130 ms, P2: 135–165 ms, and N2: 245–275). To statistically characterize potential between-group differences, these AEP amplitudes were entered into an analysis of variance (ANOVA) with group (RTT vs TD) as a between-subjects factor and stimulus-type (Tone vs Phoneme) as a within-subjects factor (IBM SPSS Statistics version 25).

It is important to note that this analysis is not sensitive to potential differences in the latencies of these AEP components, a factor we were interested in given the prior evidence for slowing of AEP responses in this group. Therefore, we conducted supplementary analyses by identifying the AEP components for each individual in the Tone condition, where the early AEP components are more pronounced and dissociable. For each individual, the P1, N1, P2, and N2 components of the AEP were identified via the following automatic procedure. P1 was estimated as the first positive component elicited at least 40 ms after onset time, N1 as the first negative component detected in the 80-200 ms latency range. The second positive component was labeled P2, and N2 was defined as the negative peak with the greatest negative-going value in the 200–400 ms range. This automatic detection algorithm corresponds well with manual peak detection. One RTT subject had no clearly detectable peaks and was excluded from further analysis. In addition, one 21-year-old TD subject had no detectable P1 component and three subjects (two TD participants aged 4.3 and 6.9 years, as well as one RTT aged 10.10 years old) had no evident N1 component. Technically, P2 could be defined in these subjects without the N1 component, but the P2 latencies deviated significantly from the rest of the sample, at least when the TD group was considered. Thus, we also excluded them from the final analysis. Student’s *t*-tests were used to examine the between-group differences in the latencies and amplitudes of AEPs in this supplementary analysis.

We were also interested in the potential of AEP components to segregate RTT individuals from those in the TD group. We therefore applied receiver-operator-characteristic (ROC)-analysis as well as a split-half reliability to evaluate internal consistency of our measure. The optimal cut-off point in ROC-analysis was assessed based on sensitivity and specificity measures^24^. Split-half reliability was assessed by calculating AEPs separately for odd and even trials and then applying similar procedures to them for measuring AEP components. Thus, in addition to the main AEP component values, each participant has two other values obtained from two-halves of the experiment. Based on these values, we calculated the Spearman-Brown split-half reliability coefficient to estimate full test reliability with the following formula: 2*Rhh/(1+Rhh), where Rhh is the Pearson correlation between measures obtained for even and odd trials. In addition, ROC-analysis was performed on split-half values to assess the stability of classification accuracy.

Pearson correlations were used to assess potential relationships between AEP components and age. We also compared the correlation coefficients for each component between groups using a Fisher z-transformation to examine if AEP maturation was similar across groups^25^. The AEP amplitudes of interest that showed between-group differences were also assessed for potential correlation with Rett Syndrome Severity Scores (RSSS) in the participants with RTT.

## Results

**Fig. 1:**
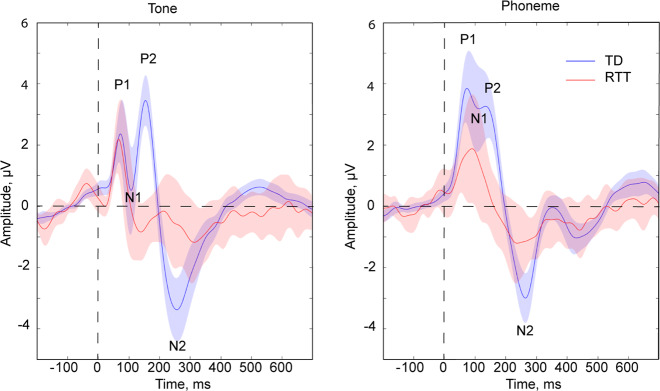
Auditory evoked potentials (AEPs) in response to Tones and Phonemes in TD and RTT groups. The vertical dashed line corresponds to the onset of the stimuli, and the horizontal dashed line indicates baseline value. Blue and red lines correspond to TD and RTT AEPs respectively. Opaque blue and red shading illustrates the standard error of the mean.

The grand-averaged AEPs in response to both tones and phonemes showed the expected pattern of identifiable P1, N1, P2 and N2 components in the TD participants (Fig. 1, single subject AEPs can be seen in Supplementary Fig. 2). This pattern was clearest for the response to the tone stimuli but was also seen in the grand mean response to the phoneme condition. AEPs in the RTT group, however, clearly deviated from those of TDs, with direct correspondence of component peaks being difficult to discern for all but the initial P1.

**Fig. 2:**
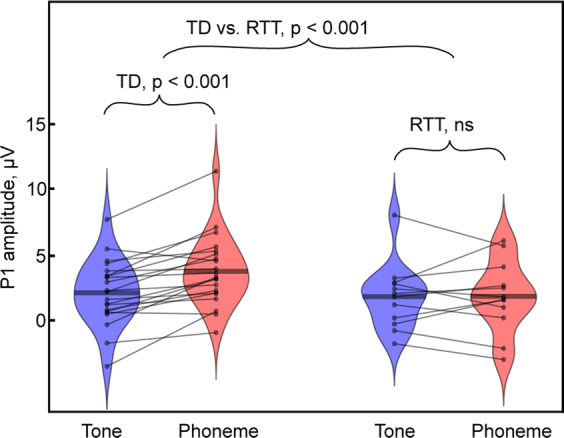
Modulation of P1 amplitude by stimulus type in TD (left) and RTT (right) groups. Each individual’s data are represented by connected dots corresponding to values in the Tone and Phoneme conditions. Note the significantly smaller P1 in the Tone than in the Phoneme condition in TD, a pattern that is not observed in the RTT group.

AEP amplitudes for each condition and group with corresponding ANOVA results are summarized in Tables 2 and 3. A significant Stimulus-type by Group interaction was observed for the P1 component, and this stemmed from the significantly greater amplitude of the P1 in response to phonemes as compared to tones (*t*(20) = 5.185; *p* < 0.0001) in the TD group, an effect that was not present in the RTT group (*t*(11) = 0.046; *p* = 0.964, Fig. 2). P1 amplitude did not differ significantly between the groups for either of the conditions. The N1 amplitude was reduced in response to phonemes compared to tone stimuli, similarly across groups, with a significant main effect of stimulus-type, but no significant Group effect nor interaction of Stimulus-type by Group.

**Table 2:** Amplitude of AEPs in children with RTT and their TD peers (mean ± SD).

	Tone, µV		Phoneme, µV	
	TD	RTT	TD	RTT
P1: 60–90 ms	2.03 ± 2.53	1.73 ± 2.57	3.70 ± 2.73	1.76 ± 2.77
N1: 100–130 ms	1.02 ± 2.71	−0.40 ± 2.24	3.22 ± 3.09	1.37 ± 3.01
P2: 135–165 ms	3.20 ± 1.73	−0.72 ± 1.50	2.94 ± 2.03	0.29 ± 1.92
N2: 245–275 ms	−3.30 ± 2.41	−0.62 ± 2.43	−2.87 ± 1.83	−1.14 ± 1.72

**Table 3:** ANOVA effects.

	Stimulus type	Group	Group X stimulus-type
P1 60–90 ms	8.996/0.005/0.225*	1.503/0.229/0.046	8.518/0.006/0.216*
N1 100–130 ms	43.026/<0.0001/0.581*	2.812/0.104/0.083	0.508/0.481/0.016
P2 135–165 ms	0.945/0.338/0.030	36.928/<0.0001/0.544*	2.681/0.112/0.080
N2 245–275	0.019/0.892/0.001	10.756/0.003/0.258*	1.614/0.213/0.049

The numbering convention lists the F-statistic value, followed by the *p*-value, followed by the effect size expressed as eta-squared (i.e. *F*/*p*/*η*2). An asterisk is used to further denote cells with significant findings.

**Fig. 3:**
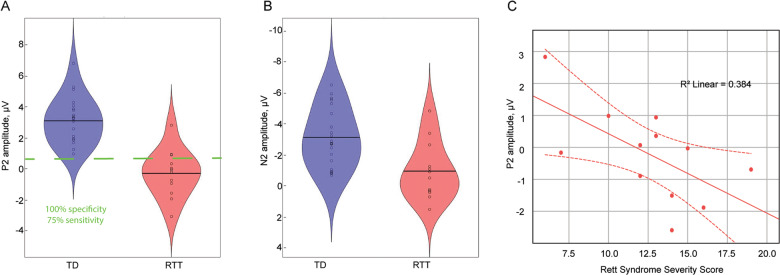
Reduction of P2 and N2 components in RTT. The figure represents data averaged across conditions (Tone and Phoneme). Each dot corresponds to an individual participant. **a** Amplitude of the P2 component in TD and RTT groups. Two short horizontal lines indicate the means of the group values. The dashed horizontal green line is drawn at the decision threshold of + 0.63 µV, where RTT and TD participants are discriminated with 100% specificity and 75% sensitivity. **b** Relationship between P2 component and Rett Syndrome Severity Scores (RSSS) in patients with RTT. Note that larger RSSS values indicate more severe symptoms. **c** Amplitude of N2 component in TD and RTT participants. Two short horizontal lines indicate the mean of each group.

In contrast to the P1 and N1, the P2 component differed substantially between the RTT and TD groups, but was not modulated by stimulus type (Table 3). As can be seen in Fig. 1, whereas there is a clear and prominent P2 component in the TD group for both Tones and Phonemes, at the group level it appears to be entirely absent in participants with RTT. The lack of statistical difference between P2 in response to Phonemes and Tones suggests that the P2 amplitudes in these two conditions should be considered together, thus we averaged P2 amplitude across conditions for further exploration. Consideration of individual subject data (Fig. 3a) reveals that only three RTT patients had P2 amplitudes exceeding the minimum value observed for TD participants (and these were still smaller than the average across TD participants). A significant Pearson correlation between RSSS and P2 amplitude suggests the relevance of the P2 component to clinical RTT manifestation (*r*(12) = −0.62, *p* = 0.032, Fig. 3b). The ROC analysis revealed an area under the curve (AUC) of 0.956 (95% CI 0.882–1.000; *p* < 0.0001). This indicates that the measured variable has very good predictability for RTT. The optimal cut-off point of +0.96 µV, allows for classification of RTT patients from controls with 95% sensitivity and 83% specificity.

The N2 response was also significantly reduced in RTT (main effect of group, Table 3, Fig. 3c). However, the result is a less robust indicator of group membership than the P2 component: only about 50% of RTT patients’ N2 amplitudes lie below the minimum N2 amplitude value seen in the TD group.

### Analysis of AEP latencies in the Tone condition

The results of this analysis confirmed the results of the primary analysis. No significant between-group differences were evident for P1 and N1 components, with their amplitudes and latencies essentially being typical in patients with RTT (*p* > 0.065). P2 and N2 components were significantly attenuated as well as delayed in patients with RTT (*p* < 0.03). However, P2 delay was much less pronounced than the P2 amplitude reduction outlined above (*p* = 0.004 vs *p* = 0.00005).

### Developmental changes in the AEP

Our study confirmed developmental decreases in P1 and N2 amplitudes and increases in N1 strength in the TD group but was not adequately powered to detect between-group differences in the developmental trajectory of AEP components (Table 4). Noteworthy, there was no significant P2 amplitude modulation with age in any group.

**Table 4:** AEP developmental changes.

	TD (*n* = 21)	RTT (*n* = 12)	TD vs RTT difference
P1 60–90 ms	−0.631/0.002*	−0.447/0.145	*z* = −0.64, *p* = 0.52
N1 100–130 ms	−0.636/0.002*	−0.183/0.569	*z* = −1.39, *p* = 0.16
P2 135–165 ms	−0.398/0.074	0.184/0.566	*z* = −1.49, *p* = 0.14
N2 245–275	0.700/<0.0001*	0.227/0.479	*z* = 1.56, *p* = 0.12

Numbers in the second and third columns are the Pearson correlation coefficients (*r*) and their significance values (*p*) for correlations between age and the amplitudes of the four AEP components of interest. Fisher’s z transformation and its significance value (*p*) were used to compare the correlation coefficients between the groups and are represented in the fourth column. An asterisk is used to denote correlations that reached significance. Note that while developmental changes were significant only in the TD group, there were no detectable differences in the correlation coefficients between RTT and TD groups.

### Scalp topographic maps

**Fig. 4:**
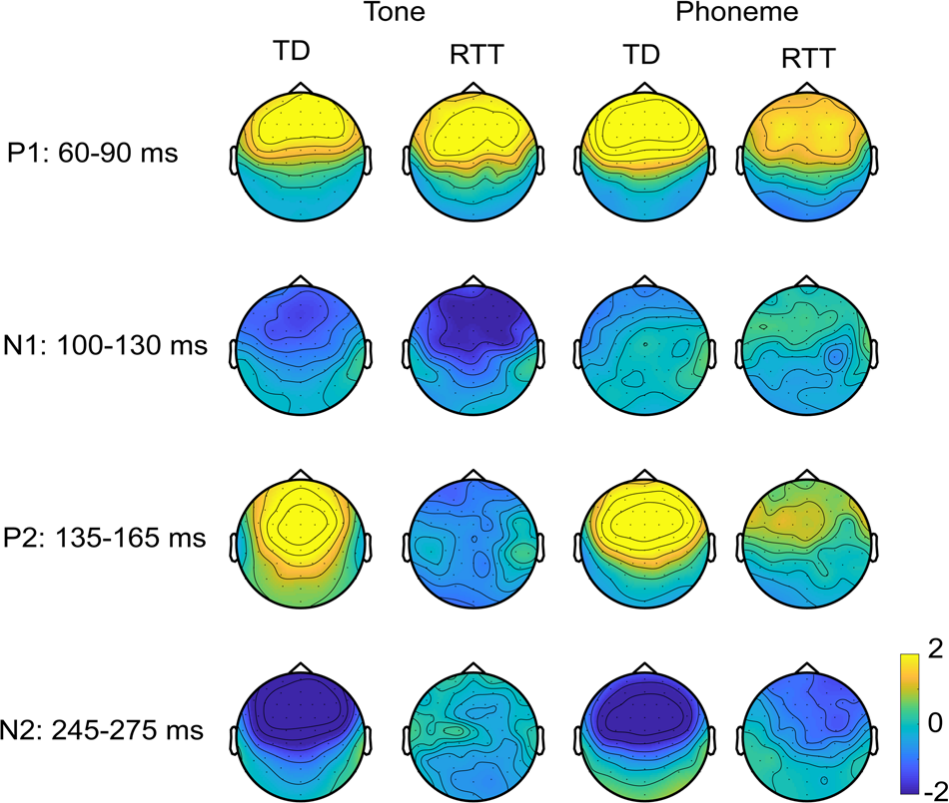
Topography of auditory evoked potentials in response to Tone and Phoneme conditions in TD and RTT groups. While topography for P1, P2, and N2 components are plotted based on averaged amplitude over corresponding time intervals, N1 topography is plotted in relation to P1 meaning that averaged over N1 latency AEP amplitudes were subtracted from AEP amplitudes averaged over P1 latency interval.

As illustrated in Fig. 4, for the TD group, topographic mapping of the tone-AEPs was consistent with a predominance of dipolar electrical activity from bilateral auditory cortex in the time-windows of the P1, N1, P2, and N2. In the RTT group, a similar scalp topography was seen for the P1 and N1 latency windows, whereas topographic mapping of the later time windows did not reveal clear topographic maps, reflecting the major drop off in signal for the RTT group following the initial cortical responses. For the Phoneme condition, the TD group again had fronto-central topographical distributions consistent with auditory cortical generators, for all but the N1 time-window. In the RTT group, for the phoneme condition, a clear fronto-centrally focused topography was present for the P1 latency window, whereas the later time windows did not show distinct topographies, again reflecting the smaller signal following the initial auditory cortical response.

## Discussion

This is the first study to examine early auditory cortical processing of complex speech stimuli in RTT, and to compare these responses to those evoked by simple frequency-specific tones. The initial AEP component (the P1 – 60–90 ms), which localizes in the region of primary auditory cortex^26–28^, showed increased amplitude to the more complex phonemic inputs relative to pure tones in TD participants. In contrast, while a relatively robust P1 component was evident in the RTT population, it did not show this modulation by stimulus type/complexity. The subsequent N1 (100–130 ms) did not differ between groups, whereas the P2 (135-164 ms), the following major deflection, was hugely diminished or even completely absent in the RTT group, regardless of stimulus type. The N2 (245–275 ms) was also substantially reduced in amplitude in RTT and showed no modulation as a function of stimulus type. The P2 effect was remarkably robust in differentiating between groups with almost perfect classification into group despite the wide age-range of our samples. Given this robustness, the P2 has potential to serve as a monitoring, treatment response or even surrogate endpoint biomarker. The implications of these findings are discussed in what follows.

The only prior study to compare early ERP components between TD and RTT groups did not observe P2 and N2 reductions in RTT, but rather, reported delayed latencies of the N1 and P2 components in response to simple tones^6^. The current data did not replicate an N1 latency delay, but confirmed a sizeable P2 delay. These discrepancies between results are likely linked to differential sample characteristics, such as sample size (7 vs 12), age range (10–22, vs 4–22) and possible differences in the genetics of the RTT participants (unknown vs confirmed *MECP2* mutation) in Baders^6^ and our studies respectively. Another contributing factor may have been lower signal-to-noise ratios in the Bader study due to considerably lower numbers of trials (*n* = 128).

### Neurophysiological differentiation of simple versus complex sounds

#### The P1 component (60–90 ms)

The P1 component, which is generated in early auditory cortex^26–28^, is known to be affected by stimulus parameters such as complexity or “speechness” in TD children^12–16^. In particular, P1 amplitude is larger in response to vowels than to simple sinusoidal tones in school-aged children^12,15^. Čeponiené et al.^15^ suggested that this increase in amplitude was related to higher neuronal synchronization in response to phonemes as compared to simple tones, whereas Bruder et al.^12^ forwarded a somewhat different explanation, positing increased P1 amplitude as a reflection of a wider neural tuning curve (i.e. larger neuronal population activation) to the phonologically richer speech sounds^12^. Here, we confirmed P1 amplification in TD children in response to spectrally richer speech sounds compared to tones, a modulation notably absent in the RTT cohort. While the P1 topographic maps for both groups and stimulus conditions were consistent with auditory cortical generators, this lack of modulation in RTT may reflect a deficit in increasing synchronization of neural response to more complex stimuli, or a breakdown in the fundamental tonotopic representation of spectral information within core auditory cortical regions. Our previous finding that the MMN to frequency deviations was both attenuated and delayed in RTT accords well with the latter interpretation, suggesting that even fundamental tonotopic representation in early auditory core regions may be weakened^8^.

#### The N1 component (100–130 ms)

The subsequent N1 component is also thought to have its major generators along the supra-temporal plane, in and around primary auditory cortex^26–29^. In contrast to P1, N1 was larger for tones than for phonemes in both groups. However, despite the apparent differences in the AEP during the N1 timeframe of 100–130 ms post-stimulus in the same\adjunct regions, our analysis did not reveal a statistically significant group difference. This may reflect greater variability at the individual participant level at this later stage of auditory processing.

### Robust RTT vs TD differentiation

#### P2 attenuation (135–165 ms)

The P2 component has been localized to several sources in auditory cortex as well as in the non-specific reticular-thalamo-cortical activating system^29–33^. A growing body of evidence has related the P2 component to consolidation processes associated with auditory memory formation and learned relevance^34^. Supporting this link, multiple studies have reported increases in P2 amplitude following perceptual learning/training^35–38^, or in populations with increased exposure to particular stimuli, such as musicians^39–41^. It is also of note that the P2 can show increased amplitude several days after initial exposure to stimuli, even when no training or additional exposure to the stimuli takes place^34,42^. This P2 augmentation to background familiar stimuli suggests that it reflects automatic integration of previous stimulus history, and does not necessitate an explicit training process *per se*. It is particularly noteworthy that a P2 increase following training or stimulus exposure, does not appear to occur immediately, but rather, is seen only on the next day, and this augmentation can then last for months^34,36,43^. This pattern of results certainly seems to suggest that the P2 is related to memory consolidation processes and that sleep plays a key role in its production. Additionally, P2 amplitude recorded during passive conditions shows an inverse relationship to behavioral improvements after training, quantified as reaction time speeding in response to the trained stimuli^44,45^. Such an association between response speed and AEP amplitude points to the relevance of P2 enhancement for effective task performance.

In the current study, the P2 component was drastically attenuated in patients with RTT, a pattern seen both in response to phonemes and tone-pips. Given the previous literature, it seems reasonable to posit that the greatly reduced P2 amplitude in RTT likely indexes severely disturbed automatic integration of current auditory input with representations from long-term memory. At the same time, automatic detection of oddball stimuli in the auditory stream is operational in this group, as evidenced by the presence of the mismatch negativity (MMN) response, albeit that this MMN is substantially delayed in latency^8^. More studies are clearly needed to investigate the functional role of P2 and its impairment in RTT.

The amplitude of this most severely affected component was below the minimal TD value in 9 out of 12 RTT subjects (75%), pointing to the clinical relevance of the P2 effect, and this clinical relevance is further bolstered by the significant correlation between P2 amplitude and the severity of Rett symptomatology, here somewhat crudely measured by the RSSS. The link between RSSS and auditory P2 might be explained by general neural abnormalities manifest in parallel in RTT. However, they might be also be linked to some specific abnormalities in particular brain regions contributing to both effects. While RSSS is an integrative measure covering multiple domains, most of these can be linked to subcortical deficits, e.g. hypotonia, breathing irregularities, sleeping problems. Taking into account that the reticular-thalamo-cortical activating system was reported to contribute to the amplitude of the P2^31^, both the clinical symptoms and P2 reduction in patients with RTT might be related to brainstem deficiencies. However, our P1 findings point to largely intact auditory inputs to auditory cortex, with subsequent auditory cortical processing in the P2 timeframe (as suggested by the scalp topographic maps) disturbed.

It is worth pointing out that P2 abnormalities have also been reported in other clinical populations. Studies in dyslexia have revealed atypical modulations of P2 by inter-stimulus interval^46^ and stimulus statistics^47^, and increased P2 has been associated with poorer reading abilities^48^. In line with what we observe here, attenuation of the P2 has been reported in idiopathic autism^49,50^, although not consistently so^51,52^. However, so far as we are aware, the substantial P2 reduction in response to auditory stimuli (64–75 dB) seen here during passive listening has not been previously reported in other neurodevelopmental conditions.

#### N2 attenuation (245–275 ms)

The following N2 component is generated in the vicinity of auditory cortex^53^, as is consistent with the topographic maps, and has potential additional sources in frontal cortex^54^. This component has been related to the inhibition of irrelevant information, as it shows attenuation with stimulus repetition^55^ in children and young adults, and it is also reduced in elderly subjects compared to younger adults^56,57^. N2 reduction has also been reported in children with developmental dysphasia^58^ and other language impairments^59^. Further supporting a relationship between N2 and language processing abnormalities, a longitudinal study found that decreased N2 amplitudes to non-speech stimuli between the ages of 4 and 8 years was associated with subsequent poorer word reading at school^60^. As such, the current finding of robust N2 attenuation in patients with RTT may index deficient inhibitory processes that are a prerequisite for typical language development.

### Effect of alertness/drowsiness on our results

Our study used a passive auditory paradigm during which participants watched a movie and were not instructed to attend the stimuli. Nonetheless, we appreciate that level of alertness can modulate AEPs and may well have differed between groups. Previous work has found that N1 is decreased while P1 and P2 are increased with the progression of sleep^61–63^. Taking this into account, the pattern of results observed in our RTT group (i.e. attenuated P2 and N2 responses, and an intact P1 response) is unlikely to simply result from decreased alertness/increased drowsiness in RTT participants. Moreover, when we generated separate average responses based on the first 150 artifact-free standard trials and last 150 artifact-free standard trials in the Tone condition, the P2 group effect was seen in both cases, and was similar to what we observed in the main analysis of all available artifact-free standard trials (see Supplementary Fig. 3). As such, this differential effect did not seem to change over the course of the experiment, during which alertness levels may have decreased.

### Bridging animal and patient studies in RTT

Since the discovery that mutations of *MECP2* were causal to Rett Syndrome, this disease has been actively studied in pre-clinical animal models with targeted genetic manipulations^64,65^. In addition to behavioral manifestations of Rett Syndrome, such as pronounced stereotypic forelimb motions, uncoordinated gait, reduced spontaneous movement, and irregular breathing, AEP components were also found to be abnormal in the rodent model of RTT^64,66,67^. Our study bridges the animal and human research by examining similar AEP components to those studied in rodent models of RTT. The most pronounced effect of P2 amplitude reduction observed in our patients was also evident in RTT animal models: P2 amplitude was reduced both in rat and mice RTT models both in response to click and speech sounds^64,66,67^. Consistent with the idea of the relevance of P2 to Rett symptomatology, P2 amplitude was typical in *Mecp2*-deficient mice before the first manifestation of symptoms^64,67^. Also in line with our results, N2 amplitude was significantly reduced in response to speech sounds in the rat model of RTT, the only model and condition where N2 was examined^66^. Noteworthy, in spite of evident attenuation of P2 and N2 components in *Mecp2*-deficient rats^66^, these rats learned to discriminate speech sounds similarly to wild-type animals. The mutant animals’ speech discrimination performance became significantly poorer than that of wild-types only in a more challenging condition that included background noise. Thus, the observed AEP abnormalities might not be crucial for simple auditory discrimination tasks but necessary for complex sound discrimination under noisy environmental conditions.

### Towards biomarker validation

Among all the current findings, the AEP amplitude within the P2 latency range (135–165 ms) showed the greatest effect size in differentiating RTT and TD groups with high ROC-characteristics. This measure also had high internal consistency as demonstrated by Spearman-Brown split-half reliability analysis. While measuring the mean amplitude within a particular latency range did not allow conclusions about potential latency shifts, this approach to measurement is advantageous in that it can be applied even when the peaks are hard to identify (e.g., whereas for the latency analysis, five participants had to be excluded due to inability to reliably identify a peak latency (15% of participants), only one participant was excluded from data analysis using this approach, and this was because of excessively noisy data). Another advantage of this measure is that it can be obtained with only a few electrodes (e.g. Cz referenced to mastoid), making it easily applicable in clinical settings. Clearly, although highly promising, considerable additional work will be needed to establish the validity and reliability of P2 amplitude as an index of Rett severity. This finding will need to be replicated in a larger sample and tested against a predefined threshold. A significantly larger sample would also allow one to differentiate RTT patients based on severity of genetic mutation, onset of symptoms and other relevant characteristics. Furthermore, ultimately one would like to see validation of P2 amplitude as an index of a particular biological process or function that could be targeted by treatment. More detailed behavioral phenotyping of the RTT sample would also be of great help. While RSSS is considered a validated clinical measure of cerebral abnormalities in RTT^68–70^ and is widely used in psychological research^17,71,72^, it is still a rather crude measure of RTT severity. And while a challenge no doubt, better characterization of auditory ability and receptive language assessment by standardized (e.g. Peabody Picture Vocabulary Test) and/or novel techniques (e.g. eye-tracking) in participants with Rett Syndrome might also be extremely helpful to link our results to specific processing deficits such as auditory speech^73^.

### Study limitations

A limitation here was the broad age-range of participants, given that the AEP continues to mature across development over the age-range tested (Table 3). Another issue was that we did not acquire hearing tests on the day of experimentation from RTT patients due to difficulties in assessing it in this population. However, the presence of prominent P1 and N1 components in the RTT group clearly indicates that the initial stages of auditory cortical processing were more-or-less intact.

Nonetheless, delayed N1, P2, N2, and P3 and reduced P2 and N2 have been associated with moderate to severe sensorineural hearing loss as reviewed in Martin et al.^74^. Clearly future research will benefit from assessing the integrity of the brainstem response and middle latency processing of auditory information in patients with RTT to determine the extent to which cortical processing atypicalities may be related to sensorineural hearing loss.

### Summary

These data provide the first in-depth analysis of the early AEP in response to speech stimuli, revealing the absence of typical modulation of the P1 component by stimulus complexity in patients with Rett Syndrome. While the next N1 component was preserved in RTT, the later AEP components, P2 and N2, were almost completely abolished. The most severely affected component, the P2, was below TD levels in most RTT participants and was associated with symptomatology. Moreover, unlike other components, P2 was stable across development. As such, the P2 AEP component holds real promise as a neuromarker of RTT. It is especially noteworthy that P2 attenuation has also been reported in animal models of RTT, providing a potentially key translational link between this neuromarker in patients and RTT animal model studies.

## Supplementary information

Supplementary materials

## Data Availability

We will make the full de-identified dataset with appropriate notation and any related analysis code available in a public repository (Figshare) and include digital object identifiers within the final text of the paper, so that any interested party can access them.
