# Supplementary material for: Atypical processing of tones and phonemes in Rett Syndrome as biomarkers of disease progression
Source: Transl Psychiatry. 2020 Jun 10;10:188. doi: 10.1038/s41398-020-00877-4 (PMC7287060; doi:10.1038/s41398-020-00877-4)

**Supplementary materials**

**Supplementary Figure 1.** Experimental stimuli. Top: Waveform of phoneme /ba/. Lower Left: Waveform of sinusoidal tone of 1000 Hz. Lower right: Spectrum of the stimuli (blue for Phoneme, red for Tone and green for the first 50 ms of Phoneme).

**
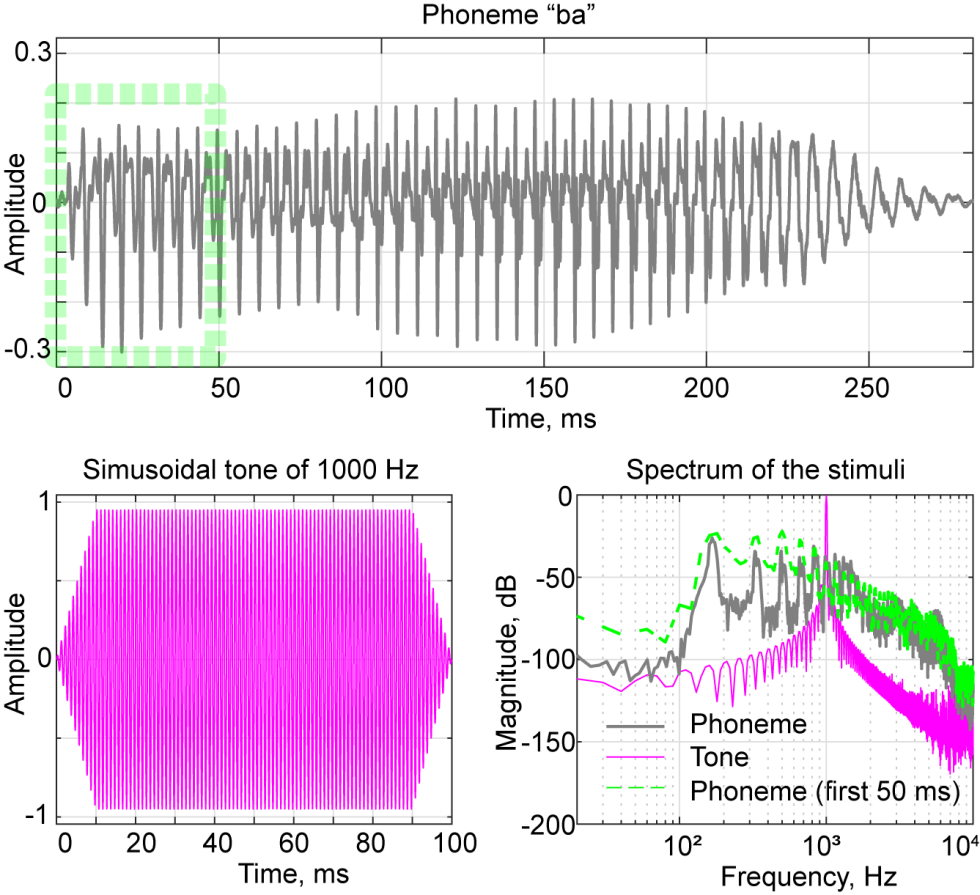
**

**Supplementary Figure 2.** Single subjects AEPs in RTT and TD participants. Each line corresponds to single participant.

**
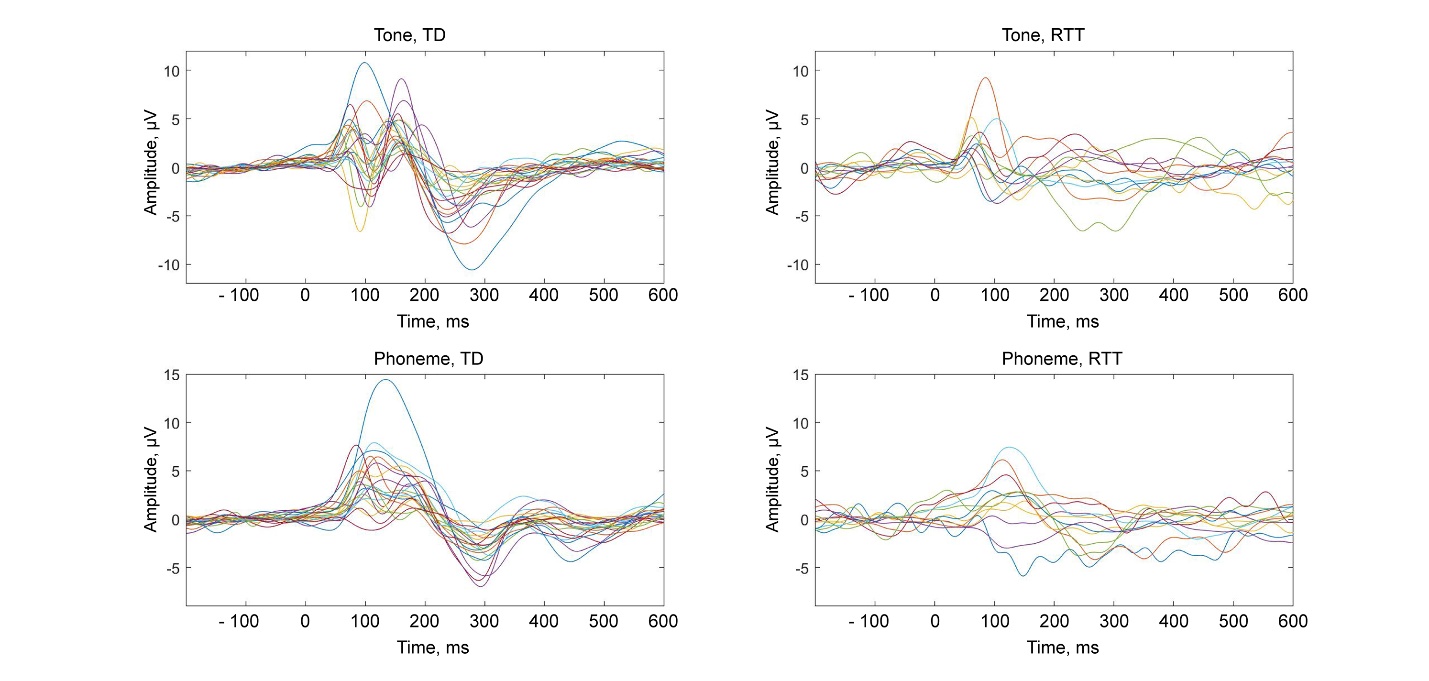
**

**Supplementary Figure 3.** AEPs from the average of the first 150 artifact-free trials and on the last 150 artifact-free trails, for the Tone condition.


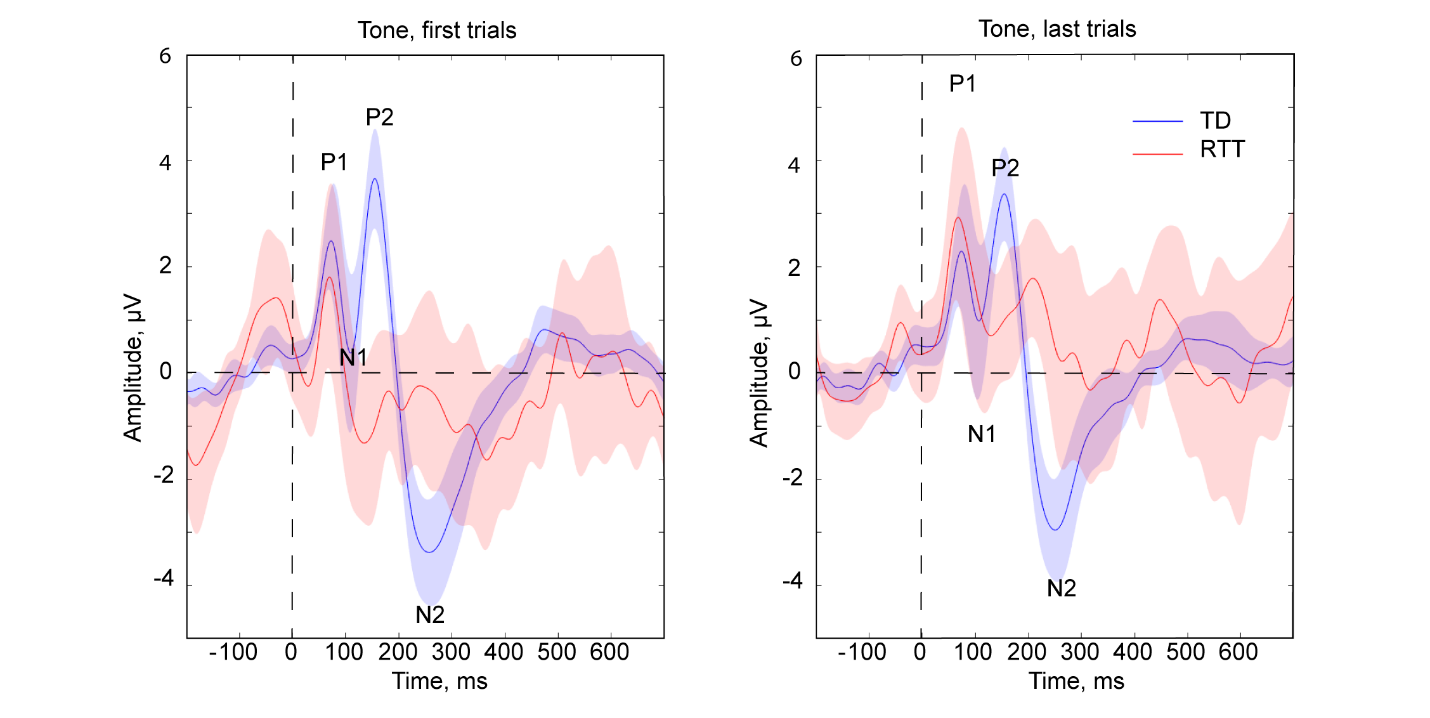

Supplement: Supplementary file 1 — Supplementary materials [file 41398_2020_877_MOESM1_ESM.docx]
